# Sensory Properties and Color Measurements of Dietary Chocolates with Different Compositions During Storage for Up to 360 Days

**DOI:** 10.3390/s90301996

**Published:** 2009-03-17

**Authors:** Jovanka V. Popov-Raljić, Jovanka G. Laličić-Petronijević

**Affiliations:** University of Belgrade, Faculty of Agriculture, Belgrade, Nemanjina 6, Serbia; E-Mail: jovankal@agrif.bg.ac.rs (J.G.L.P.)

**Keywords:** Dietary chocolates, sensory attributes, color measurement, storage

## Abstract

In this work sensory characteristics (appearance – color, brilliance, shape and surface; texture – structure, break, firmness and chewiness; aroma – odor and taste) of dietary chocolates of different compositions were evaluated, in parallel with color parameter measurements. Color was determined instrumentally on the top and bottom surfaces, using a “MINOLTA” Chroma meter CR 400 thristimulus colorimeter. Sensory evaluation was performed by a group of experienced panelists immediately after the production (0 – 30 days), and then after 90, 180, 270 and 360 days of storage under ambient conditions (t = 18 – 20°C). Results were statistically analyzed by the two-factorial analysis of variance (MANOVA) and with the LSD – test. It was concluded that the storage time up to one year had statistically highly significant (p < 0.01) effects on the sensory attributes of chocolate, as well as on instrumentally measured color parameters.

## 1. Introduction

The objective of this research was to determine color quality characteristics by instrumental measures and evaluate sensory properties of dietary chocolates, regarding theirs different compositions as well as duration of storage.

Dietary chocolates represent a special kind of the exceptionally high quality chocolates. Their raw materials could be chosen for the purpose of increasing or decreasing their energetic values. During the estimation of quality of chocolates, besides the determination of chemical and physical parameters, the sensory quality must be evaluated (i.e. appearance – color, flavor, texture), immediately after the production and during their storage. Sensory properties of chocolate are considered to be among the most important parameters when defining general chocolate quality. General sensory acceptance or a customer’s likeability are key factors for successful placement of a chocolate on the market. Chocolate is consumed by consumers mainly for pleasure, i.e. enjoyment, and, far less, for its nutritive value.

The literature provides a lot of data concerning the problems of sensory definition of the changes in quality of different chocolate types, which are narrowly related with the possibilities of applying individual raw materials and definite additives (compositions) at particular phases of the processing and storage processes [1–13]. During storage, chocolate surfaces turn grayish (“fat bloom”), inducing considerable color changes, i.e. of lightness, nuance and saturation of color. The turning grey of chocolate, principally, appears as the result of errors during defined phases of the technological production processes, such as tempering, forming, cooling, or as a consequence of extremely long storage [4,14–17].

In the competent literature, opinions were expressed to the effect that main reasons for chocolate turning grey during storage are temperature fluctuations and inadequate tempering conditions, inducing migration of fats through the matrix of chocolate particles, that follows their recrystallization on the surfaces. The loss of brilliance and emersion of turning grey are the consequences of dissipation of light on the “clusters” of crystals [18–21].

Brilliance is an important quality parameter of chocolate and it is a key factor for tempering control [21]. Brilliance is an optical phenomenon connected by the appearance and represents ability of the surface to reflect a direct light [22]. There exists an opinion that the turning grey of chocolate represents a development of a new phase in the fatty phase of the chocolate, which appears as the result of the polymorphous transformations of the fourth crystal form into the fifth, i.e. into the sixth one [23]. Therefore, if the chocolate is stored at temperatures below 15°C, it is possible to inhibit these polymorphous transformations [24].

Changes of chocolate surface colors are mainly evaluated sensorially (using visual techniques), or by colorimetric or spectrophotometric instrumental measurement methods [9,25–28]. The whiteness index (WI) could be used as one of the parameters of the defining of color quality characteristics (whitening of chocolate surface), which is, most probably, a consequence of color changes induced by inadequate conditions during equalization of chocolate temperature, after the cooling phase, as well as any inadequate storage conditions [29].

## 2. Materials and Methods

### 2.1. Materials

During storage of dietary chocolates after 0 – 30, 90, 180, 270 and 360 days, two sets of experiments have been carried out in parallel:

1. analysis of color blooming by means of CIE and CIEL*a*b* detection by an instrumental method (Minolta CR 400 colorimeter)
2. measurement of sensory properties of the chocolates using experienced panelists.

Dietary chocolate samples of different compositions produced under industrial conditions were used. The chocolate samples had the following compositions:

Sample 1 - Fructose, cocoa butter, whole milk powder (19%), cocoa mass, skimmed milk powder, hazelnut paste, emulsifier (lecithin), aroma. Cocoa content at least 35%.
Sample 2 - Fructose, cocoa butter, inulin, skimmed milk powder, cocoa mass, milk fat, hazelnut paste, emulsifier (soy lecithin), aroma, table salt. Cocoa parts at least 35%, milk parts at least 14%.
Sample 3 - Fructose, cocoa mass, whole milk powder, cocoa butter, inulin, hazelnut mass, emulsifiers (soy lecithin, polyglycerol, polyricinolate), vanillin aroma. Cocoa parts dry basis at least 30%, fat-free dry substance at least 10.5%.
Sample 4 - Sweetener lactitol (E966) as sugar substitute, cocoa butter, dry milk powder, whey powder, cocoa mass, sugar substitute polydextrose (E1200), hazelnuts, emulsifier lecithin (E322) sweetener aspartame (E1200) vanillin aroma. Cocoa parts at least 32%, milk fat at least 3.65%.
Sample 5 - Cocoa butter, sweetener lactitol (E966), whole milk powder, polydextrose, skimmed milk powder, cocoa mass, emulsifier (E322), sweetener aspartame (E951), sweetener acesulfame potassium (E950), ethyl aniline, aroma. Total dry substance of cocoa parts at least 27%. Total milk dry substances at least 28%, milk fat at least 4%.

### 2.2. Color Measurements

During the 1st month from the declared production time, i.e. in the period from 0 – 30 days, and after 90, 180, 270 and 360 days of storage at the appropriate room temperature (t =18–20°C) color was measured instrumentally with a MINOLTA CR 400 Chroma meter thristimulus photoelectric colorimeter, with a D light source, standard 65° viewer and 8 mm light beam diameter. Color was measured at three predetermined places of the chocolate top and bottom surfaces (n = 50). Results were expressed in the CIE system, as the average values: Y = average reflectance or brilliance; λ= dominant wavelength (nm), and color purity P(%) [30].

In the CIE system, average reflectance or brilliance is determined on the basis of the Y (%) – value which is readed out directly at the Minolta CR-400.

**Figure 1.**
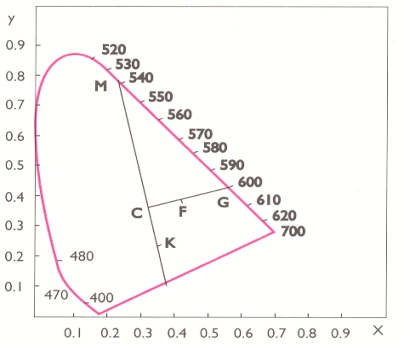
Determination of dominant wavelength and purity of color by CIE system.

Dominant wavelength (λ) is determined on the basis of the calculated trichromatic coefficients, which are introduced into the chromacity diagram (point F), which is to be jointed with the point C, and extender to the intersection with the spectral curve. Point of intersection [point G represents the dominant wavelength (Figure 1)].

Color purity is expressed in percents and it is to be calculated on the basis of the following relation of points:

(1) $$\text{color purity}\;(\%)\;P=\frac{C\cdot F}{F\cdot G}\cdot 100$$

In CIEL*a*b* results were given as the mean values: L* – psychometer light, a* – psychometer tone and b* – psychometer chroma [31–34]. Color quality characteristics are expressed on the basis of the following equations:

(2) $$\text{psychometric light}\;L*=116\cdot {\left(\frac{Y}{Y_{0}}\right)}^{\frac{1}{3}}-16$$

(3) $$\text{psychometric tone}\;a*=500\cdot \left[{\left(\frac{X}{X_{0}}\right)}^{\frac{1}{3}}-{\left(\frac{Y}{Y_{0}}\right)}^{\frac{1}{3}}\right]$$

(4) $$\text{psychometric chroma}\;b^{*}=200\cdot \left[{\left(\frac{Y}{Y_{0}}\right)}^{\frac{1}{3}}-{\left(\frac{Z}{Z_{0}}\right)}^{\frac{1}{3}}\right]$$

a* - psychometric tone [participation of red (+) and green (–) colors of components];
b* - psychometric chrome [participation of yellow (+) and blue (–) colors of components].

Whiteness Index (WI) was calculated according to [20,28]:

(5) $$\text{WI}=100{\left[{\left(100-L*\right)}^{2}+{\left(a*\right)}^{2}+{\left(b*\right)}^{2}\right]}^{0.5}$$

For determination of degree of difference of color between sample and the standard white, ΔE^*^-values were calculated [32]:

(6) $$\Delta E^{*}=\sqrt{\Delta L^{*2}+\Delta a^{*2}+\Delta b^{*2}}$$

### 2.3. Sensory Analysis

Sensory properties of the dietary chocolates were followed during the whole period of storage, using all known relevant ISO standards [35–39].

Sensory evaluations include the selected, representative, or dominant attributes of chocolate qualities. Irrespective of the number of ascribed points, or of scoring span, each score has to be described with words. Namely, Planck [40] as far back as 1947, introduced and scientifically explained the so-called “weight coefficient”. With application of the weight coefficients, a quantitative expression of the total product quality is obtained as the “weighted” mean value of the scores for each the evaluated parameter. Because of that, before of performing the evaluations, it is important to determine weight coefficient for each property, and balance them in such a way, that their sum equals to 20 (Table 1).

### 2.4. Statistical Analysis

Data obtained in the investigations performed in this study were analyzed by descriptive and analytical statistics. Basic parameters of the descriptive statistics included calculations of the arithmetic mean values, and variability parameters of the investigated properties included determinations of standard deviations (Sd) and variation coefficients (Cv) expressed in percents.

For analytical statistics (for evaluation of sensory determinations data of the chocolates), the two-factorial analyze of variance MANOVA was applied, with the first factor being the storage time, and the second one – the composition of the evaluated chocolate samples, as well as the LSD-test (test of the least significant differences of pairs).

For finding out if the prerequisites for variance analysis methods are justified, homogeneities of variances were determined using Levene’s test. For the data which, based on the Levene’s test, showed homogenous variances, parameter statistics was applied, and in the case of inhomogeneity of variances, the parameter statistics was applied because that, for the applied two-factorial experiment it was not possible to apply the nonparametric statistics [Statistics – V.6-package] [41,42].

In the instrumental determinations of characteristics of color measurement, the trend equation best fitting the average reflectance (Y, %) changes was obtained with the statistical software Origin 6.1 (Origin Lab. Corporation, Northampton, MA, USA).

## 3. Results and Discussions

### 3.1. Color Measurements of Different Dietary Chocolate Samples

**Figure 2.**
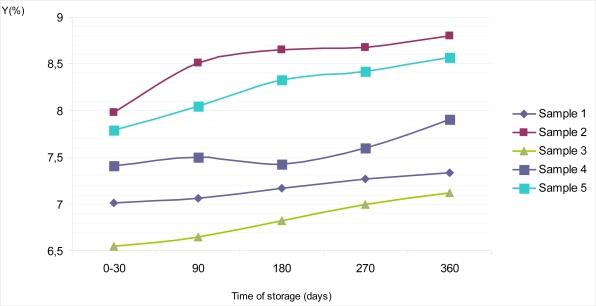
Changes of average reflectance (Y, %) on the top surface of samples of dietary chocolates during storage of 360 days.

**Figure 3.**
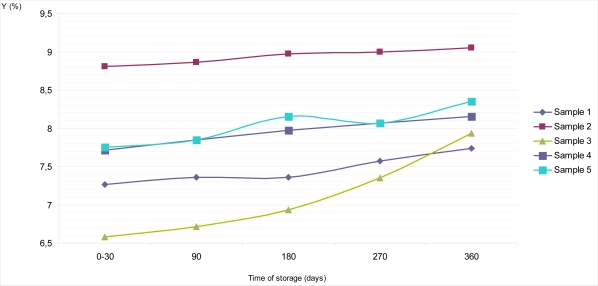
Changes of average reflectance (Y, %) on the bottom surface of samples of dietary chocolates during storage of 360 days.

**Figure 4.**
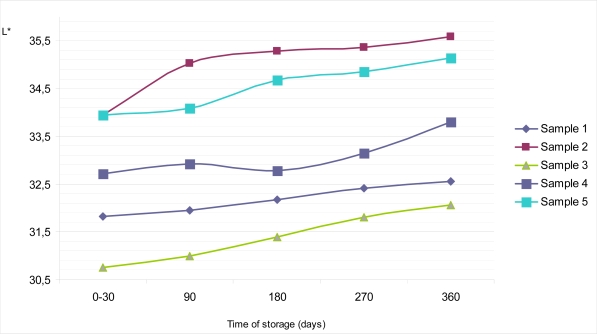
Changes of psychometric light (L*) on the top surface of samples of dietary chocolates during storage of 360 days.

**Figure 5.**
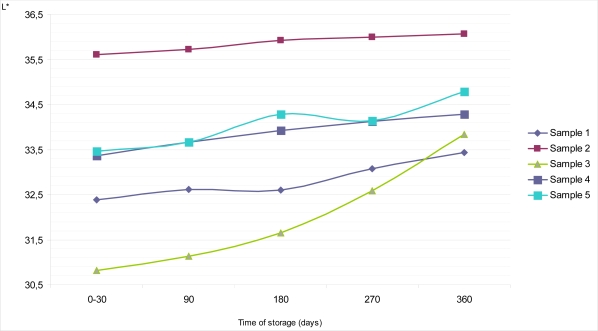
Changes of psychometric light (L*) on the bottom surface of samples of dietary chocolates during storage of 360 days.

Average reflectance or brilliance on the top surface of Sample 1 during the period of 0 – 30 days of storage at of 18–20°C was Y = 7.01%, with S_d_ = 0.70 and C_v_ = 0.07. During further storage it barely grew, so after 360 days of storage Y equals 7.34% with S_d_ = 0.70 and C_v_ = 0.07 (Figure 2). Similar features could be observed for psychometric light in the CIE L*a*b* system. Thus, the value of L* amounted to 31.83 at the very beginning of examinations, and after one year it was L* =32.56 (Figure 4). If the values obtained for an average reflectance Y (%) and lightness L*, in the CIE L*a*b* system were compared with the same values obtained for the dietary chocolates’ colors measured on the bottom surfaces, it is evident that these values for bottom surfaces were slightly higher (Y =7.26% with S_d_ = 0.70 and C_v_ = 0.07 in a period of 0 – 30 days and Y = 7.74% with S_d_ = 0.70 and C_v_ = 0.07 on the 360^th^ day) (Figure 3). Lightness during the interval went from L* = 32.39, immediately after production, up to L* = 33.43 (Figure 5). The observed values for dominant wavelength during the whole period of storage were approximately similar on the top, as well as on the bottom surfaces, so that values of λ = 590 nm were observed for the very 1st measurement, and λ = 588 nm on the 360th day of examination. According to the chromaticity diagram, these both values belong to the same region of spectrum – the orange one. During storage, values of psychometric tone (a*) and chroma (b*) decreased. Color purities (color saturations) of all samples were similar; for 270 days of storage were found to be P = 29.59%, and after a year they decreased to P = 26.32%.

Based on the obtained results, dietary chocolate Sample 2 was the sample having the lightest color. Namely, its brilliance on the top surface of chocolate was in the interval between Y = 7.98% and Y = 8.80%, with the lightness values of L* = 33.95 to L* = 35.59, all in the frames of storage up to one year (Figures 2 and 4). This was also confirmed by the calculated values for dominant wavelengths, which, according to the chromaticity diagram belonged to the yellowish – orange part of spectrum (λ = 588 nm). Psychometric tone (a*) slowly decreased during storage, but the participation of chroma (b*) had been subjected to negligible growth for the top, as well as for the bottom chocolate surfaces. In the period between days 0 and 30 the mentioned parameters amount to a* = 12.85 and b* = 14.12, and after one year they were only insignificantly higher (a* = 12.95; b^*^ = 14.22).

Sample 3 was relatively “the darkest one” according to the color nuance. Starting from the 0th, and up till the 360th day, values of the average reflectance for the top chocolate surface seemed to be the lowest, compared with all other dietary chocolate samples analyzed, having values from Y = 6.55% to Y = 7.12%, i.e. L* = 30.76 to L* = 32.07 (Figures 2 and 4). Psychometric tone (a*) during storage decreased, chroma (b*) slowly grew and color saturation (λ = 590–591 nm) remained approximately the same and belonged to the orange part of spectrum. Lightness of the dietary chocolate 3, measured on the bottom surface, amounted from Y = 6.58% to Y = 7.93%, i.e. L* = 30.82 to L* = 33.84; therefore, it showed a mild growth tendency during one year of storage (Figures 3 and 5).

The analyzed dietary chocolate Sample 4 was, with respect to the color characteristics qualities, most similar to Sample 1, but, values for samples of the former (Sample 4) were a little bit lighter than for Sample 1. Therefore, for the top chocolate surface it had values of Y = 7.41% (S_d_ = 0.70; C_v_ = 0.09) in the period from 0th to 30th day, and Y =7.91% (S_d_ = 0.70; C_v_ = 0.09) after 360 days of storage (Figure 2). Psychometric tone (a*) during storage decreased, from a* =13.88 to a* = 12.92 in the CIE Lab system, but the values of b* grew (from b* = 13.07 to b* = 13.43). The dominant wavelength was in interval λ = 591–592 nm, and, based on the chromaticity diagram, it belonged to the orange part of the spectrum. Lightness of Sample 4 on the bottom surface was a little bit lighter than on its top surface. In a period 0 – 30 days, average reflectance on the bottom surface was somewhat lighter than on its top surface. In a period 0 – 30 days, average reflectance was Y =7.71% (S_d_ = 0.70; C_v_ = 0.09), and on the 360th day Y = 8.15% (S_d_ = 0.70; C_v_ = 0.09) (Figure 3). At the same time, the corresponding values of psychometric light were L* = 33.37 and L* = 34.29, respectively (Figure 5).

Dietary chocolate Sample 5 possessed uniform standard color quality, both on the top, and on the bottom surfaces, as follows from the average reflectance (Y, %) results in the CIE system, as well as from the observed dominant wavelengths for characterization color nuance and color saturation (purity). In the 0 – 30 days period, lightness on the top surface of this chocolate was Y = 7.98%, with L* = 33.94 (Figures 2 and 4). If these samples were kept and stored for up to one year, mild growth of these parameters was observed, so that the corresponding values were Y = 8.05%, L* = 34.09 (after 90 days), Y = 8.33%, L* = 34.67 (after 180 days), Y = 8.42%, L* = 34.85 (after 270 days) and finally Y = 8.57%, L* = 35.14 (after 360 days) (Figures 2 and 4).

The obscure differences appear at the instrumental measurement of color quality of the dietary chocolate Sample 5, on the bottom surface of chocolate (Figures 3 and 5). That the analyzed sample had really uniform, standard color quality was also confirmed by the totally identical values of dominant wavelengths for the top and bottom surfaces of this sample during its storage for up to one year at 18–20°C (λ = 590 nm – orange part of spectrum). Identical values for color saturation, i.e. color purity, P = 29.59%, and practically uniform shares of red (a*) and yellow (b*) pigments during the whole period of investigations at constant temperature, were also calculated.

**Figure 6.**
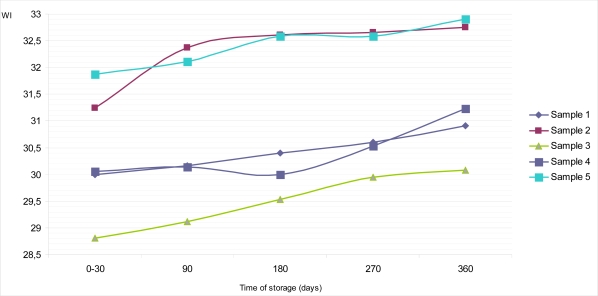
Changes of whiteness index (WI) on the top surface of samples of dietary chocolates during storage of 360 days.

**Figure 7.**
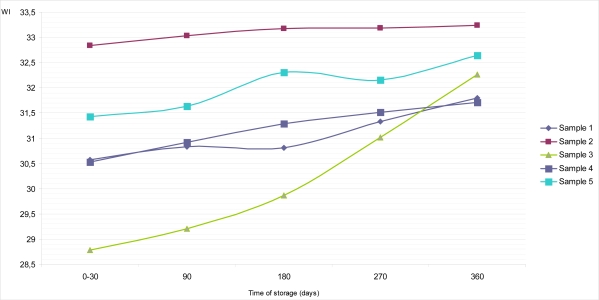
Changes of whiteness index (WI) on the bottom surface of samples of dietary chocolates during storage of 360 days.

The calculated values of whiteness indexes (WI) of all samples of dietary chocolates for their top and bottom surfaces, during up to 360 days of storage at temperature of 18–20°C, are given in Figures 6 and 7. Sample 5 had the highest values of WI on the top surface, both at the beginning (31.87) and at the end (32.90) of examination, compared to all other samples. Also, a similar situation was noticed with Sample 2, which had supreme values on the bottom surface (32.84 for period 0–30 days; and 33.24 for period 360 days of storage). On the other hand, Sample 3 maintained its lowest WI values on the top surface during whole period of research (28.81 for period 0–30 days; 30.08 for period 360 days).

Generally, with the exception of Sample 5, we could conclude that the colors estimated at the bottom surfaces of dietary chocolates (Samples 1, 2, 3 and 4) were slightly lighter than those estimated on top surfaces (Figures 6 and 7), and that was confirmed with the whiteness index data. These results are consistent with the literature data [15,29], indicating to the appearance of gray – whitish (turned grey) chocolate. However, as the turning grey depends of the microstructure of chocolate formed during the production process and further on, during storage, color changes, *inter alia*, are specific for each kind of chocolate [19].

**Figure 8.**
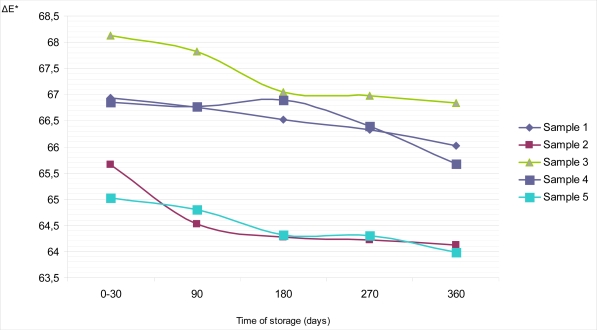
Changes of color difference (ΔE) on the top surface of samples of dietary chocolates during storage of 360 days.

**Figure 9.**
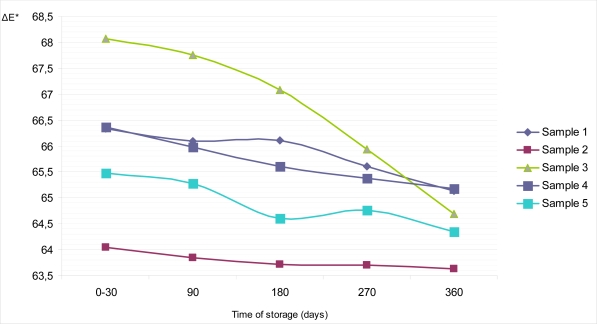
Changes of color difference (ΔE) on the bottom surface of samples of dietary chocolates during storage of 360 days.

In addition, the total color difference (ΔE), parameter which describes a colorimetric difference between two objects, for all samples during whole storage time was calculated. Figures 8 and 9 show the changes of ΔE value with time, on the top and on the bottom surface of dietary chocolates. It could bee seen that values of ΔE are rather equalized during storage, with exception of bottom surface of sample 3, where appreciable differences are noticed (68.07 for period 0–30 days; 64.69 for 360 days of storage).

Considering all presented results, during storage of samples of dietary chocolates of different compositions at 18–20°C and up to one year, it is possible to confirm claims of other authors, who have stated that composition, and the particular phases of technological process of production, as well as the inappropriate and/or too long storage are the primary influences on the characteristics of color quality [4,14–17]. Having in mind that the analyzed chocolate samples were kept and stored at constant temperature, up to one year, it could be assumed that polymorphous transformations during storage were prevented [24].

Table 2 shows statistical model of average reflectance (Y) changes (directly taken from the MINOLTA photoelectric thristimulus colorimeter) of examined samples of dietary chocolates against their time of storage (x) for up to one year.

The obtained results indicate that there exists a high correlation for values of average reflectance during storage, with values of correlation coefficient in range of 0.89 to 0.99 for all analyzed samples, other than for Sample 4 (value of correlation coefficient is 0.85 on the top surface).

### 3.2. Sensory Evaluations of Different Dietary Chocolate Samples During Their Storage for up to One Year

For obtaining objective sensory evaluation results spaces for pretreatment of samples, for sensory panel work and an office for the evaluation leader are essential [35–39]. Observations registered with senses serve for the acceptance, treatment, evaluation and interpretation of impressions obtained in the sensory analysis procedure. It is most important to define three basic sensory properties: appearance (sight sense) consistency/texture (sight sense – touch sense – taste sense) and aroma (odor and taste sense).

**Figure 10.**
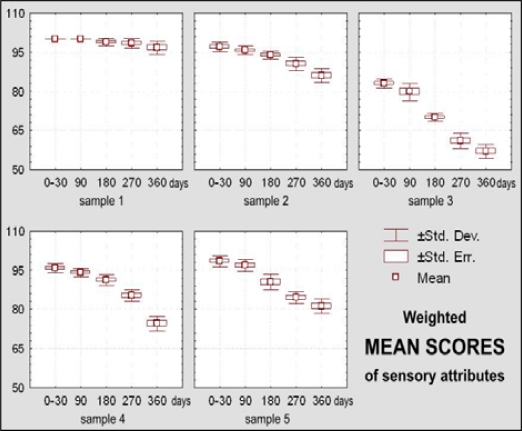
Box-plots for weighted mean score for evaluated sensory attributes of dietary chocolate.

**Figure 11.**
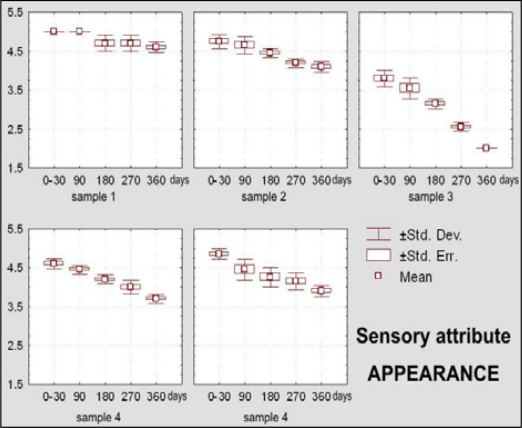
Box-plots for sensory attribute appearance of dietary chocolate.

**Figure 12.**
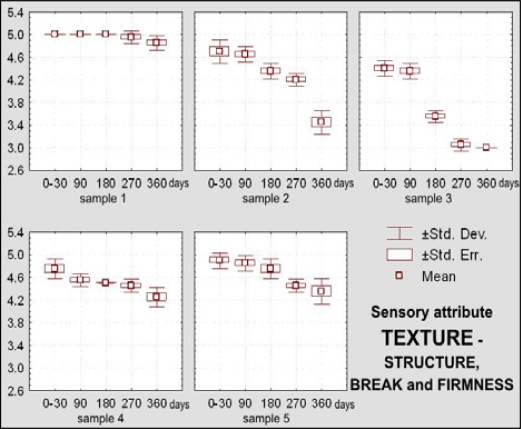
Box-plots for sensory attribute texture-structure, break and firmness of dietary chocolate.

**Figure 13.**
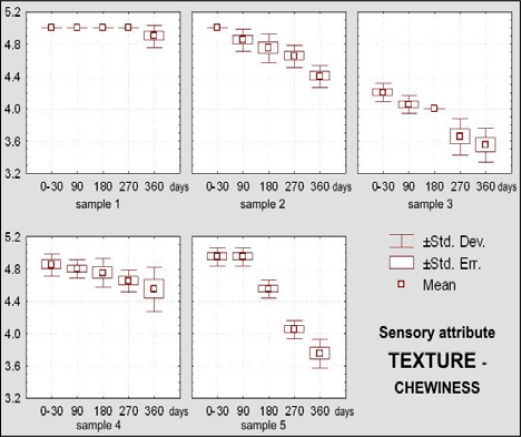
Box-plots for sensory attribute texture – chewiness of dietary chocolate.

**Figure 14.**
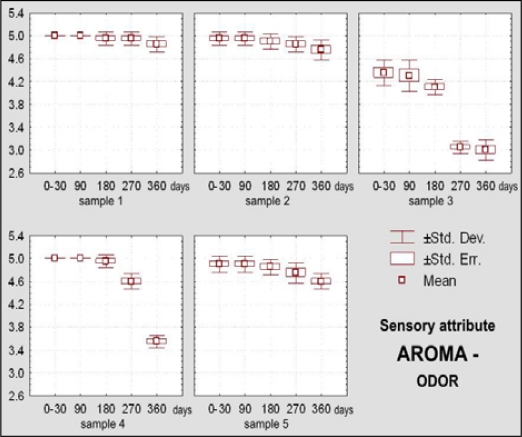
Box-plots for sensory attribute aroma – odor of dietary chocolate.

**Figure 15.**
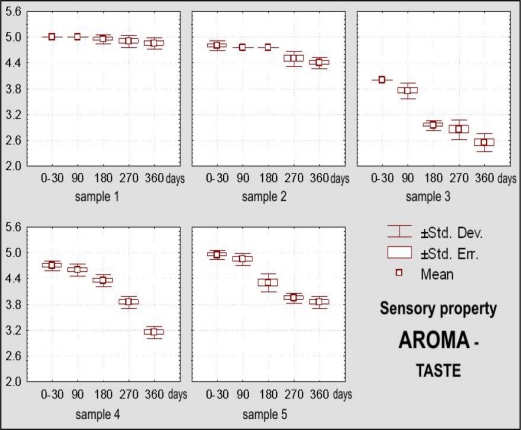
Box-plots for sensory attribute aroma – taste of dietary chocolate.

Sample 1 kept its excellent general sensory quality during 270 days of storage (Figure 10). At 360 days insignificant appearance changes were observed (X_m_ = 4.60) (Figure 11), as well as negligible changes of texture and aroma (Figures 12–15).

The dietary chocolate identified as Sample 2 had excellent sensory quality during the first month after production (X_m_ = 4.85 or 97% of the maximal possible quality), which was retained for a period of 270 days (X_m_ = 4.30, or 85.95% of the maximal possible quality) (Figure 10). Among sensory quality parameters, the highest changes were observed for texture; namely, visual evaluations of structure revealed the appearance of small-grained structure, followed with oral changes of chewiness described as sandy character (Figures 12 – 13).

Dietary chocolate Sample 3 had relatively good sensory quality during storage. Immediately after production it was already evaluated with a very good mean score (X_m_ = 4.15, or 83% of the maximal quality). After 180 days of storage, sensory quality of this chocolate sample was just between very good and good (X_m_ = 3.50, or 70% of the maximal quality) and after that period it was assigned a category of good quality, with 2.85, or only 57.05% of the maximal possible quality (Figure 10). This sample was evaluated with the lowest initial score (X_m_ = 3.80) for appearance as a sensory parameter, and after 360 days it was evaluated as unacceptable (X_m_ = 2.00) (Figure 11), primarily because of existence of stains at its top surface. These stains were blue-greenish in color so that, besides the appearance of the fat bloom, there existed a possibility of some effect of the packaging. On the bottom surface of this chocolate notches and prominences appeared. Also, borders were worn-out and crumbs with the presence of “sawdust” were noted. All these features had negative effects with respect of general quality of the chocolate. Besides, panelists observed changes of texture, i.e of break structure and firmness, absence of typical chocolate odor and presence of taste with a distinctive salty note.

Dietary chocolate Samples 4 and 5 had during the first 180 days of storage excellent sensory qualities, and after 360 days it was very good. Sample 5 was scored with higher marks (from initial X_m_ = 4.92, namely 98.45% during 0–30 days, to 4.06, namely 81.20% after 360 days) than the Sample 4 (from X_m_ = 4.79, namely 95.75% during 0–30 days, to X_m_ =3.73, namely 74.60% after 360 days) (Figure 10). Sample 4 was characterized with more prominent changes of appearance (lighter and nonhomogenous color of the top surface) (Figure 11), texture (larger grains and sandy impression on chewing) (Figures 12 and 13), as well as some changes of taste (Figure 15). In spite of the higher scores for Sample 5, which retained its characteristic color during the whole examination period, separation of the fat phase on the top and bottom surfaces and appearance of a quantity of “sawdust” caused definite lower scorings for the appearance as sensory attribute. In addition, some changes of chewiness (slower melting in mouth) (Figure 13) and less pronounced odor were registered for the sample (Figure 14).

It should be noted that although the samples of dietary chocolates were sweetened either with sugar substituents, or with different sweeteners with different degrees of sweetness with respect to sucrose, the general conclusion of panelists was that these substances, as well as their blends are properly balanced, meaning that neither enhanced nor lesser sweetness was noticed compraed to standard milk chocolates. However, because all monosaccharides, including fructose, and also sugar alcohols that include lactilol, have somewhat different taste profiles compared with sucrose, it is possible to expect the appearance of an after-taste, which can be manifested in forms of scratching or “burning” in the throat. The mentioned effects were observed for Samples 3 and 4, and that type of statements could be found in the literature as well [43]. One of possible reasons for the somewhat different taste decribed for Sample 5, in comparison with other samples, could be the use of acesulfame potassium and of aspartame sweeteners, the applied formulations, or the applied technological procedures [1–4,6,8,11].

During sensory evaluations of texture, appearing of sandiness on chewing and visually large-grained or fine-grained structures were observed in the dietary chocolate samples. In order to overcome such effects the introduction of distinctive changes to the technological processes applied to these types of chocolates was suggested [43], consisting of separate conching of milky chocolate mass and sugars or sugar supplements, sugar alcohols and sweeteners, which results in better taste and removal of moisture from the chocolate mass, thus inducing disappearance of sandiness and of grainy structures.

Results for Levene’s test for homogeneity of variances of samples of dietary chocolate samples (Samples 1, 2, 3, 4 and 5, Table 3) that indicate homogeneity for weighted mean scores; for aroma - odor, the data are inhomogeneous, while attributes of appearance, texture, and aroma – taste were very inhomogeneous.

According to results of two-factorial variance analysis for the group of dietary chocolate Samples 1, 2, 3, 4 and 5 (Table 4), it is evident that both the observed factors – duration of storage and the different composition as well as their interaction – have statistically very significant effects on all examined sensory quality attributes (p < 0.01).

Based on the results of LSD test for sensory attribute *appearance* of dietary chocolates, it can be stated that this attribute changes statistically very significantly in all periods of storage during one year of time. Also, between declared compositions of samples there exist differences on the significance level of p < 0,01 with exception that between declared compositions of Samples 2 and 5 differences are significant at the level of p < 0.05.

Visual attributes (appearance) include observations on sensory properties of food products that are registered by the sight sense (eyes), and they are shape, color, surface and similar [44]. Appearance of chocolate includes following visual attributes: brightness, color, shape, roughness, surface texture, turbidity and other. Appearance, according to Briones and Aguilera [18], depends on complex interaction of the incident light, its optical properties and visual sensory perception. Figure 11 outlines box-plots for sensory attribute *appearance* of dietary chocolate Samples 1, 2, 3 4 and 5.

Sensory attribute *texture,* which includes visually evaluated structure and break, palpatory evaluated firmness and orally evaluated chewiness of dietary chocolates was not statistically significantly changed (p > 0.05) during first 90 days of storage, and after that period differences were statistically highly significant (p < 0.01). Texture (structure), break and firmness of dietary chocolates with different compositions were statistically highly significant (p < 0.01). Differences of chewiness are also statistically highly significant, with exception of compositions of Samples 2 and 4, where statistical significance of differences was not confirmed (p > 0.05).

Complexity of texture is a sensory attribute that depends on the food structure (molecular, microscopic and macroscopic), as well as irreplaceability of human beings for description of this multiparametric attribute, which is perceived with several senses, confirming the data of Szczesniak [45]. Figure 12 shows texture-structure, break and firmness changes, and Figure 13 – chewiness changes of dietary chocolate Samples 1, 2, 3, 4 and 5 during their storage.

Sensory evaluated attribute of *aroma – odor* was not statistically significantly changed during 90 days of storage. After 180 days differences for smell are at the significance level p < 0.05, and after 270 and 360 day they are on the significance level p < 0.01. Between Samples 1 and 2, differing according their compositions, statistically significant differences for the considered attribute do not exist, but differences between Samples 2 and 5 are statistically significant (p < 0.01). The remaining samples showed statistically significance differences of odor in respect of their composition.

The sense of odor characterizes basic sensory information for estimation of quality and intensity of odor, and besides to that, of taste, what is of the primary importance for determination of aroma [46]. Figure 14 shows changes of the aroma – odor of Samples 1, 2, 3, 4 and 5 during their storage for up to one year.

*Aroma – taste* is statistically highly significantly (p < 0.01) during storage for up to one year. The same show results obtained with the LSD test. Between Samples 1, 2, 3, 4 and 5 of different compositions there exist significant differences with respect to their taste.

Figure 15 shows changes of the aroma – taste of dietary chocolate Samples 1, 2, 3, 4 and 5 during their storage for up to one year. Based on the LSD test it could be stated that the weighted mean score of the dietary chocolate samples changed at the significance level of p < 0.05 after a period of 90 days, while after 180, 270 and 360 days of storage these changes were at the significance level p < 0.01. Influence of different composition reveals that there exist statistically highly significant differences with respect to the weighted mean score between samples of dietary chocolates during whole period of storage. Figure 10 shows changes of the weighted mean scores for all evaluated sensory attributes of Samples 1, 2, 3, 4 and 5 during their storage for up to one year.

## 4. Conclusions

- According to results obtained by the two-factorial analyze of variance – MANOVA, composition, storage time, as well as their interaction have statistically very significant (p < 0.01) influence on sensory attributes of chocolate quality (appearance – color, brightness, shape and surface; texture – structure, break, firmness, chewiness; aroma – odor and taste).
- In the segment of sensory analysis of dietary chocolates, Sample 1, which consisted of fructose, cocoa butter, whole milk powder (19%), cocoa mass, skimmed milk powder, hazelnut paste, and lecithin as emulsifier, along with added aromas, was evaluated sensorily as excellent during storage up to one year, with values of X_m_ of 5.00, namely 100% of the maximal quality (0–30 days of storage) to 4.83, namely 96.70% of the maximal quality (360 days of storage). On the other hand, Sample 3 which consisted of fructose, cocoa mass, whole milk powder, cocoa butter, hazelnut mass, vanillin aroma, with addition of inulin and combination of emulsifiers (soy lecithin, polyglycerol, polyricinoleat), during storage up to one year demonstrated striking transformations of appearance, texture and aroma, hence in the last storage period (after 360 days) the value for X_m_ was 2.85, namely 57.05% of the maximal quality (near the sensory acceptability limit).
- Results of instrumental measurements of color of top and bottom surfaces of dietary chocolates, obtained with the thristimulus colorimetry (in CIE and CIE L*a*b* systems) are in good agreement with sensory evaluations of color and brightness of chocolate samples, so that they enable precise estimations of even the lowest color changes, that almost cannot be noticed visually, if evaluations are not performed by very experienced and trained panelists.

## Figures and Tables

**Table 1.** Sensory evaluation of the chocolate quality using the scoring procedure

**Basic sensory properties**	**Score**	**Weight coefficient**	**Description of the evaluated property**
APPEARANCE Form, color, brightness, surface	5	2	Appropriate form; irreproachable color; smooth, bright surface; clear print
	4		Insignificant deviation of form; irreproachable color; smooth, bright surface; print less clear
	3		Deviations of form; lower quality color; fingerprints on the surface; air bubbles; print less clear
	2		More pronounced form deviations; partially white of gray surface; presence of cuttings
	1		Form distorted; surface gray or white; higher damages; print bad
TEXTURE Structure, break, firmness	5	3	Break straight, homogenous, fragile; structure homogenous; texture smooth; firmness appropriate
	4		Break uneven; structure homogenous; firmness appropriate
	3		Break uneven, air bubbles; firmness inappropriate; fat bloom appearance on the break
	2		Break uneven; texture roughly-granular; fat bloom on the break
	1		Crumbling; texture roughly granular; fat bloom
Chewiness and other textural properties	5	4	Appropriate chewiness; melting in the mouth
	4		Slower melting; good chewiness, spreadiness
	3		Average chewiness; spreadiness; weak sandiness
	2		Slow melting; sandiness; stickiness
	1		Slow melting; heavy sandiness; stickiness
AROMA	5	4	Appropriate; rounded; aromatic
	4		Appropriate poorer rounded; aromatic
	3		Appropriate; poor rounded; weakly aromatic
	2		Not appropriate; sourish; staled
	1		Foreign odor; sour; staled; mouldy
Odor	5	7	Appropriate; rounded; aromatic
Taste	4		Appropriate, less rounded; aromatic
	3		Poorly rounded; poorly aromatic
	2		sourish; not rounded
	1		Foreign taste; sour; bitter

*Quality category* was determined in dependence of scores spans; products, which were evaluated with less than 2.5 points, were considered as unsatisfactory, i.e. as inacceptable; scores within limits 2.5 – 3.5 characterized good quality products, 3.5 – 4.5 very good quality and 4.5 – 5 – excelent products.

**Table 2.** Statistical model of the average reflectance (Y) changes for analyzed dietary chocolate samples during their storage for up to 1 year.

**Mathematical model:**	**Y = a+ bx**			**Correlation coefficient**

**Sample No.**	**Surface**	**Parameters**		
		**a**	**b**	

1	Top	6.99	0.001	0.99**
	Bottom	7.77	0.001	0.95*

2	Top	8.16	0.002	0.89*
	Bottom	8.81	0.001	0.98**

3	Top	6.53	0.001	0.99**
	Bottom	6.43	0.003	0.96**

4	Top	7.35	0.001	0.85
	Bottom	7.73	0.001	0.99**
5	Top	7.96	0.002	0.98**
	Bottom	7.75	0.001	0.94*

**Table 3.** Results of the Levene’s test of homogeneity of variances of dietary chocolate.

**Sensory attribute**		**Levene’s test**	
		**F**	**p**

Appearance		3.411	0.000

Texture	Structure, break and firmness	2.716	0.000
	Chewiness	3.617	0.000

Aroma	Odor	1.930	0.013
	Taste	3.362	0.000

Weighted mean scoring value		1.469	0.097

**Table 4.** Results of two-factorial analysis of variances of dietary chocolate.

**Sensory attribute**		**Storage time**		**Composition**		**Interaction**	

		**F**	**p**	**F**	**p**	**F**	**p**

Appearance		128.018	0.000	412.378	0.000	8.986	0.000

Texture	Structure, break and firmness	142.319	0.000	326.486	0.000	20.757	0.000
	Chewiness	75.446	0.000	233–679	0.000	10.463	0.000

Aroma	Odor	118.776	0.000	311.474	0.000	27.674	0.000
	Taste	213.479	0.000	605.521	0.000	23.091	0.000

Weighted mean scoring value		219.471	0.000	591.447	0.000	16.904	0.000
